# Non-Bismuth Quadruple Therapy, Sequential Therapy or High-Dose Esomeprazole and Amoxicillin Dual Therapy for First-Line Helicobacter pylori Eradication: A Prospective Randomized Study

**DOI:** 10.7759/cureus.11837

**Published:** 2020-12-02

**Authors:** Meriem Zeriouh, Amine Elmekkaoui, Mouna Bouqfar, Abdelkrim Zazour, Wafaa Khannoussi, Ghizlane Kharrasse, Naima Abda, Zahi Ismaili

**Affiliations:** 1 Gastroenterology and Hepatology, Mohammed VI University Hospital Center/Mohammed First University, Oujda, MAR; 2 Gastroenterology and Hepatology, Mohammed VI University Hospital Center, Oujda, MAR

**Keywords:** helicobacter pylori, eradication, non-bismuth quadruple therapy, sequential therapy, dual high dose therapy

## Abstract

Aim: The study aims were to evaluate and compare the effectiveness and safety of non-bismuth quadruple therapy with sequential therapy and dual therapy with high dose esomeprazole and amoxicillin as an empirical first-line approach to eradicate *Helicobacter pylori* infection.

Patients and methods: Prospective randomized trial included 393 patients infected with *H. pylori* naïve to eradication therapy, randomized to receive a 10-day non-bismuth quadruple or concomitant (CT) therapy, 10-day sequential therapy (SQ), or 14-day high-dose esomeprazole and amoxicillin (BT). Treatment outcome was assessed by C13-urea breath test at least six weeks after therapy. Adverse events and compliance were assessed with questionnaires and residual medication count.

Results: The baseline demographic clinical and endoscopic characteristics were similar among the three groups. The intention to treat (ITT) analysis was performed in 130, 132, and 131 patients in the BT, SQ, and CT groups, respectively. The eradication rates in ITT were 64.6%, 83.1%, and 92.3%, respectively, in the BT, SQ and CT groups (p = 0.0001). The eradication rates per protocol were 67.7%, 88.5%, and 95.3% (p = 0.0001), respectively, in the BT, SQ, and CT groups. The CT and SQ groups were higher than the BT group (p = 0,0001) but no significant results were seen in the eradication rate between CT and SQ, both in PP analysis and in ITT analysis (p = 0.09). The prevalence of the side effects following the non-bismuth quadruple therapy was 38.2%, significantly higher (p = 0.001) than the BT group (13.80%) and SQ group (22%). There were no significant differences in compliance among the three therapies (p = 0.16).

Conclusion: This study found that non-bismuth quadruple therapy yielded a higher *H. pylori* eradication rate over sequential regimen as a first-line treatment in Morocco, with no statistical difference between the two protocols studied, while the eradication rate of dual high-dose of esomeprazole and amoxicillin did not exceed 60%. All three therapy schemes showed excellent compliance. However, the prevalence of side events was more important and significantly higher with non-bismuth quadruple therapy.

## Introduction

*Helicobacter pylori *infection is one of the most widespread infections in the world. It infects more than half of the human population globally and plays an important role in the genesis of peptic ulcer disease and other digestive pathologies, notably, gastric malignancy and gastric mucosa associated lymphoid tissue lymphoma [1-4]. Its prevalence in Morocco is estimated at 70% [5].

The increase in bacterial resistance (especially for clarithromycin) over time around the world makes the treatment of *H. pylori* a challenge [6]. The Maastricht V/Florence Consensus recommends non-bismuth quadruple or concomitant therapy as first-line empirical *H. pylori* treatment (proton-pump inhibitors (PPI), amoxicillin, nitroimidazole and clarithromycin) for 10 to 14 days, in order to achieve an eradication rate of over 90% [7]. However, sequential treatment formerly recommended by Maastricht IV is still prescribed in our country with higher eradication rates compared with triple therapy and is well-tolerated [8-10].

On another note, high-dose dual therapy consists of administration of both amoxicillin (≥2.0 g/day) and PPI more than two times daily for 14 days, first introduced in the mid-nineties, has reported greater efficacy (over 90%) in recently published studies [11-15]. This regime can keep the intragastric pH at a value higher than 6.5 regardless of CYP2C19 genotype and thus maintain an observed plasma concentration of amoxicillin upwards the minimal inhibitory concentration for *H. pylori,* achievable by higher doses and frequency of PPIs [16-18].

To date, no study has compared and assessed the efficacy among all these therapies. Therefore, we decided to conduct this first randomized controlled trial, to evaluate and compare the effectiveness, safety and compliance of non-bismuth quadruple therapy, sequential and a high-dose dual therapy as an empirical first-line approach to eradicate *H. pylori *infection.

## Materials and methods

Patient population

This prospective randomized trial, about 393 patients, was conducted at the department of Hepato-Gastroenterology Mohammed VI University Hospital Oujda, Morocco, between July 2016 and September 2018. Written informed consent was obtained from all participants before the eradication treatment is prescribed. Newly diagnosed patients infected with *H. pylori *naive to treatment were included in our study. The presence of *H. pylori* infection was established by one of the following averages: 13C-urea breath test or histology. Exclusion criteria were: previous *H. pylori* eradication therapy, known allergic history to any of the medications used in our study, use of PPI or antibiotics in the previous four weeks, previous surgery of upper gastrointestinal tract, severe diseases (cardiovascular, pulmonary, renal or hepatic), a malignant disease during the previous five years, addiction to alcohol or illicit and recreational drugs or severe psychiatric or neurologic disorders, pregnant or breastfeeding women, age of <18 years and refusal to consent.

Study design and intervention

The participants were randomly assigned to one of the following treatments using a computer-generated list and with the use of a full-dose of esomeprazole 40mg twice daily as the PPI: the BT group received high-dose dual therapy comprising PPI and amoxicillin 1g 3*/d for 14 days; the SQ group received sequential therapy consisting of administration of PPI + amoxicillin 1g twice daily for five days followed by PPI + clarithromycin 500mg and metronidazole 500mg twice daily for five days; and the CT group received non-bismuth quadruple therapy, PPI + amoxicillin 1g + clarithromycin 500mg + metronidazole 500mg twice daily for 10 days. The treatment was well explained to all participants. At the end of the treatment, adverse events and compliance to therapy were evaluated. Compliance was determined by pill counts at the follow-up visit. Pill intake rate greater than 90% was considered as good compliance. Side effects were assessed using a structured questionnaire.

Six weeks after the end of the treatment a C13 urea breath test (UBT) was performed to assess *H. pylori* eradication rate. Use of the PPI, H2 receptor blocking agent and antibiotics were not allowed for four weeks before the urea breath test. A negative result of C13 UBT was considered as successful *H. pylori* eradication. In case of eradication failure (defined as a positive result of C13 UBT), patients received a second-line therapy.

Statistical analysis

We calculated the sample size needed before the start of the study based on available data in the literature by the assumption of an eradication rate of 90% for non-bismuth quadruple therapy, sequential and for high-dose dual therapy, and to detect a difference of 15% in the eradication rates between the three drugs therapy groups with a power of 80%. A 10% maximum lost to follow-up has been estimated. Therefore, the final sample size calculated was 393 patients (at least 130 patients per group) with margin of error of 5%.The results of this study were analyzed on an intention-to-treat (ITT) population and a per-protocol population with 95% CI each treatment group. The ITT analysis included all randomized patients who had taken at least one dose of the study medication. Per protocol included only patients who had made a correct follow-up and a compliance of a minimum of 90% of each study drug. For all other variables, the Fisher’s exact test and Student’s t test were used. Statistical analyses were performed through Statistical Package for Social Sciences (SPSS) version 21 (IBM Corp., Armonk, NY, USA). A p-value of < 0.05 was deemed statistically significant.

## Results

Patient population

**Figure 1:**
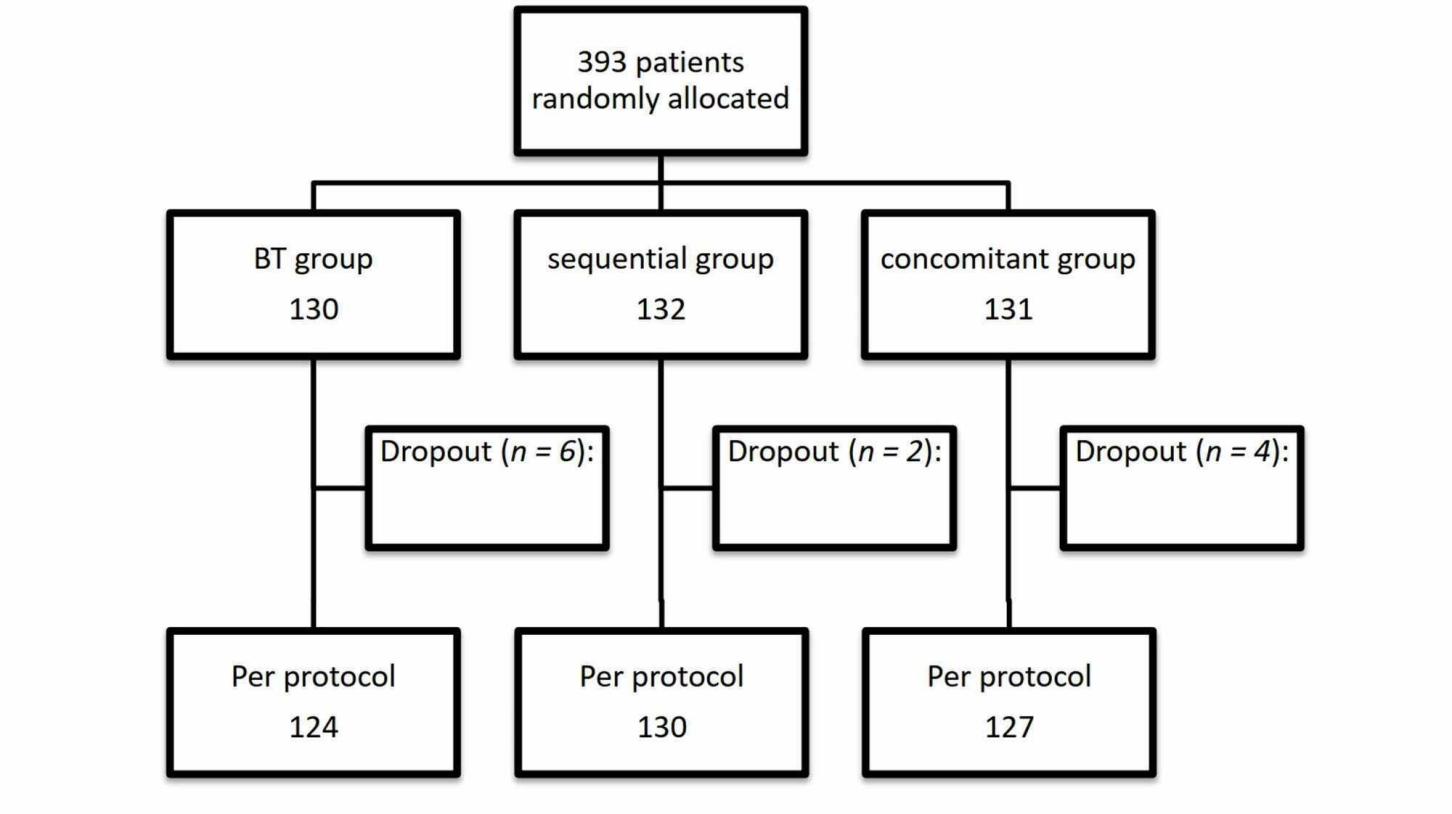
Flow diagram of the study

A total of 393 patients agreed to participate in the study (analyzed in an ITT protocol), randomized in three treatment groups (non-bismuth quadruple therapy VS sequential treatment VS high dose dual therapy), 131 received concomitant treatment, 132 sequential treatment and 130 high-dose dual therapy. Of the 131 CT patients, four had not done the breath test after the end of treatment, two of 132 in the SQ group, and six in the BT group. However, the study population available for the final per-protocol (PP) analysis consisted of 127, 130, and 124 patients in the CT, SQ, and BT groups, respectively (Figure 1).

Regarding clinical and endoscopic characteristics, there were no significant differences between the different therapeutic groups (Table 1).

**Table 1:** Baseline demographic clinical and endoscopic characteristics of the study patients SD, standard deviation; BT, high-dose esomeprazole and amoxicillin; SQ, sequential therapy; CT, concomitant therapy

	BT group N = 130	SQ group N = 132	CT group N = 131	p
Age (mean ± SD)	41.73±14	46.3±16	43.8±14	0.05
Sex ratio F/M	1	1.3	1	0.2
Tobacco	18% (n = 23)	14.4% (n= 19)	22% ( n= 30)	0.2
Alcohol consumption	10.8% (n= 14)	9% (n= 12)	13.7% (n= 18)	0.5
Endoscopic findings				0.6
Peptic ulcer dyspepsia	32.3% (n= 42)	36.4% (n= 48)	37.4% (n= 49)	
Bulbar ulcer	21.9%	22%	19.7%	
Gastric ulcer	6.4%	8.4%	8.5%	
Gastric/bulbar sores	4%	5.8%	9.2%	
Non ulcerative dyspepsia	67.7% (n= 88)	63.6% (n= 84)	62.6% (n= 82)	0.6
Normal endoscopy	2.3%	1.9%	3.2%	
Gastritis	61.8%	57%	55.3%	
Esophagitis	3.6%	4.7%	4.1%	

Eradication rate

The eradication rate in ITT was 64.6%, 87.1% and 92.3% respectively in the BT, SQ and CT groups (p = 0.0001). The eradication rate of eradication by per-protocol was 67.7%, 88.5% and 95.3% respectively in the BT, SQ and CT groups (p = 0.00016). The eradication rate obtained with non-bismuth quadruple therapy was significantly higher than that obtained with dual therapy, both in the ITT (92.3% vs 64.6%,p = 0,0001) and PP (95.3% vs 67.7%, p = 0.0001) analysis. Also the*H. pylori* eradication rate achieved with the sequential regimen was significantly higher than that obtained with the dual protocol, both in ITT (87.1% vs. 64.6%, p < 0.0001) and PP (88.5% vs. 67.7%, p < 0.0001) analysis. However, the eradication rate of the CT group was higher than the SQ group both in ITT and PP analysis and there were no significant results seen in the eradication between these protocols (p = 0.09), and both of them were higher than the BT group (p = 0.0001). The eradication rates of the three protocols by ITT and PP analysis are shown in Table 2.

**Table 2:** Treatment efficacy CI, confidence interval; ITT, intention to treat; PP, per-protocol; BT, high-dose esomeprazole and amoxicillin; SQ, sequential therapy; CT, concomitant therapy

	BT group (N=130)	SQ group (N=132)	CT group (N=131)	p
Eradication rates (%) in the ITT analysis 95% CI	64.6% (84/130)	87.1% (115/132)	92.3% (121/131)	0.0001
	BT group (N=124)	SQ group (N= 130)	CT group (N=127)	
Eradication rates (%) in the PP analysis 95% CI	67.7% (84/124)	88.5% (115/130)	95.3% (121/127)	0.0001

Compliance and adverse events

The overall adherence rate among these 393 patients was more than 90% and there was no significant difference between treatment groups (BT: 93.8% vs. SQ: 98.5% vs CT: 96%, p = 0.16). The rate of adverse reactions following the non-bismuth quadruple therapy was 38.2%, significantly higher than the BT group (13.80%) and SQ group (22%) (p = 0.001). The most common adverse events were diarrhea, affecting 57 patients (14.5%), followed by abdominal pain in 37 patients (9.4%) and metallic taste in 35 patients (9%). All side effects disappeared shortly after the end of treatment (Table 3).

**Table 3:** Side effects and compliance ^ > 90% of a medication was taken BT, high-dose esomeprazole and amoxicillin; SQ, sequential therapy; CT, concomitant therapy

Adverse events	BT group (N=130)	SQ group (N= 132)	CT group (N= 131)	p
Diarrhea	7% (9)	12.9% (17)	23.7% (31)	0.001
Abdominal pain	3.1% (4)	10.6% (14)	14.0% (19)	0.005
Metallic taste	2.3% (3)	4.5% (6)	19.8% (26)	0.0001
Nausea/Vomiting	0.8% (1)	3.8% (5)	10% (13)	0.001
Headache	3.1% (4)	3.8% (5)	8.4% (11)	0.11
Skin eruption	1.5% (2)	0.8% (1)	0.8% (1)	0.7
Asthenia	3.1% (4)	3.8% (5)	4.6% (6)	0.8
Compliance^	93.8%	98.5%	95.9%	0.16

## Discussion

The purpose of the present prospective randomized trial was to evaluate and compare the effectiveness and safety of non-bismuth quadruple therapy versus sequential therapy versus high-dose dual *H. pylori* eradication therapy, in order to achieve an ITT cure rate of at least 90% for the three protocols.

Regarding efficacy, our results showed that non-bismuth quadruple therapy cured more than 90% of treatment-naïve patients and revealed a cut superiority of this regimen over the sequential and high-dose dual therapy as first-line treatment, both in the ITT and in the PP analysis (92.3% versus 87.1% versus 64.6% p = 0.0001, and 95.3% versus 88.5% versus 67.7%, p = 0.0001, respectively). Moreover, there was no difference in the eradication rates of non-bismuth quadruple therapy and sequential therapy (p = 0.09) and both of them were higher than the BT group (p = 0.0001).

These results are in concordance with several studies and meta-analyses comparing the concomitant versus sequential treatment regimen [19-22]. Notably, there was no significant difference between concomitant regimen compared with the sequential regimen.

In Wu et al. [20], who used therapeutic regimens similar to those prescribed in our study, using full-dose esomeprazole-based PPI 40 mg twice daily demonstrated higher*H. pylori *eradication rates with both protocols (concomitant and sequential therapy) (approximately 90%).

The *H. pylori* eradication efficacy for the non-bismuth quadruple therapy and sequential treatment presented in our study also agree with those reported in the latest meta-analysis published in 2015 by He et al. and Kim et al. [21,22], respectively, suggested that both non-bismuth quadruple therapy and sequential regimen achieve equivalent, and high eradication rate. In addition to this, in a recent multicenter, prospective study by Chung et al. including 517 patients proved a similar result, an equal eradication rate between empirical 10-day sequential and concomitant therapy (ITT: 70.6%, vs 77.8% / PP: 89.5% vs 94.4%, respectively) [19].

However, these results contradict those of the last meta-analysis by Wang et al., published in 2018 and including 20 randomized clinical trials (RCTs) [23]. The efficacy of non-bismuth quadruple therapy was duration dependent, in other words, the *H. pylori* eradication rate of 10-day sequential therapy was superior to that of five-day concomitant therapy (82.09 versus 77.79%, relative risk (RR) 1.052 (95% confidence interval (CI) 1.004-1.103), p = 0.035)), similar to that of seven-day concomitant therapy (82.40 versus 86.99%, RR 0.959 (95% CI 0.874-1.053), p = 0.382). Whereas 10-day concomitant therapy was superior to 10-day sequential therapy (83.32% versus 78.39%, RR 0.945 (95% CI 0.907-0.984, p = 0.006).

Generally, antibiotic resistance is the main factor in the failure to eradicate *H. pylori*. For that, the other option was high-dose dual therapy consisting of amoxicillin and esomeprazole to eradicate *H. pylori* infection. This therapy was proposed to evaluate as first-line therapy. Firstly because the resistance of *H. pylori* to antibiotics has considerably increased in most countries [24], secondary, primary resistance to amoxicillin is very low all over the world [25]. Indeed, amoxicillin has a bactericidal effect against *H. pylori.* It depends on an intragastric pH of 5.5 or higher, and it is more stable at a higher intragastric pH achievable by higher doses and frequency of PPIs [18] and avoidance of acidic foods [26]. In our country, the prevalence of primary resistance to amoxicillin (AMO), metronidazole (MTZ), and clarithromycin (CLA) were about 0%, 40.1% and 28.6% respectively [27].

Several multicenter trials conducted in Germany in the 1990s showed that dual therapy containing a high dose of omeprazole (120 mg per day) was an effective first-line treatment due to high *H. pylori* eradication rates between 82% and 95% [11,12]. In addition, a recent study conducted in Taiwan demonstrated that a high-dose dual therapy of rabeprazole 20 mg (four times a day) and amoxicillin 750 mg (four times a day) for 14 days resulted in an eradication rate of 95.3% in naïve patients [13]. Another recent study by Sapmaz et al. documented that a 14-day protocol with rabeprazole 20 mg (three times a day) and amoxicillin 750 mg (three times a day) has an acceptable *H. pylori* eradication rate of 84.7% by ITT analysis, and it has similar efficacy when compared with bismuth-containing quadruple therapy [14]. Similarly, in Zullo et al. study’s, performed in Italy, 10-day, high-dose dual therapy with esomeprazole and amoxicillin could achieve high eradication rates with an overall eradication of 87.5% (95% CI=78.8-96.2) [15].

However, these results were in conflict with our finding. The eradication rate for the high-dose dual therapy was lower than those of the CT and SQ therapy with a rate of 60% (BT vs CT+ SQ, p = 0.0001). The reason for the different results may be the wide variations in antibiotic resistance prevalence between countries.

Regarding compliance, all three therapy schemes showed excellent compliance, which joins the results found in the literature [23].

Nevertheless, the non-bismuth quadruple therapy group had a much higher incidence of side effects than sequential and BT treatments. The higher efficacy and also the safety of the CT regimen may be related to the use of three antibiotics at the same time. The prevalence of side effects of CT protocol was 38.2% versus 22% and 13.8% with the ST and BT (p = 0.0001) respectively. Our results differ from those reported in the 2018 meta-analysis [23], which found no significant difference in the total number of side effects. However, in our trial, we observed that diarrhea was statistically significantly more frequent in the non-bismuth quadruple therapy group (CT=23.7% versus SQ=12.9% versus BT=6.0%, p = 0.001), this is in concordance with the last meta-analysis by Wang [23], which showed that diarrhea was more frequent with non-bismuth quadruple therapy than with sequential treatment. Whereas these side effects did not cause nonadherence to treatment.

The main limitation of our trial is not evaluating antibiotic resistance in the studied patients. Secondarily, this is a single medical center study.

## Conclusions

In summary, we found that non-bismuth quadruple therapy or concomitant regimen yielded a higher *H. pylori *eradication rate over sequential regimen as a first-line treatment, with no statistical difference between the two protocols studied. While the eradication rate of high-dose dual therapy of esomeprazole and amoxicillin did not exceed 60%. All three therapy schemes showed excellent compliance. However, the prevalence of side events was more important and significantly higher with non-bismuth quadruple therapy.
